# Identification of a Common Different Gene Expression Signature in Ischemic Cardiomyopathy

**DOI:** 10.3390/genes9010056

**Published:** 2018-01-22

**Authors:** Yana Li, Qiu Jiang, Zhiwen Ding, Guijian Liu, Peng Yu, Guoliang Jiang, Ziqing Yu, Chunjie Yang, Juying Qian, Hong Jiang, Yunzeng Zou

**Affiliations:** 1 Shanghai Institute of Cardiovascular Diseases, Zhongshan Hospital and Institutes of Biomedical Sciences, Fudan University, 180 Fenglin Road, Shanghai 200032, China; 15111210014@fudan.edu.cn (Y.L.); jiang.qiu@zs-hospital.sh.cn (Q.J.); DN020704@163.com (Z.D.); agreman@163.com (G.L.); 15111210028@fudan.edu.cn (P.Y.); jgltj@163.com (G.J.); 15111210029@fudan.edu.cn (Z.Y.); yang.chunjie@zs-hospital.sh.cn (C.Y.); qian.juying@zs-hospital.sh.cn (J.Q.); 2 Shanghai Institute of clinical bioinformatics, Zhongshan Hospital, Fudan University, 180 Fenglin Road, Shanghai 200032, China; 3 Institutes of Biomedical Sciences, Fudan University, 130 Dong’an Road, Shanghai 200032, China

**Keywords:** ischemic cardiomyopathy, microarrays, bioinformatical analysis

## Abstract

The molecular mechanisms underlying the development of ischemic cardiomyopathy (ICM) remain poorly understood. Gene expression profiling is helpful to discover the molecular changes taking place in ICM. The aim of this study was to identify the genes that are significantly changed during the development of heart failure caused by ICM. The differentially expressed genes (DEGs) were identified from 162 control samples and 227 ICM patients. PANTHER was used to perform gene ontology (GO), and Reactome for pathway enrichment analysis. A protein–protein interaction network was established using STRING and Cytoscape. A further validation was performed by real-time polymerase chain reaction (RT-PCR). A total of 255 common DEGs was found. Gene ontology, pathway enrichment, and protein–protein interaction analysis showed that nucleic acid-binding proteins, enzymes, and transcription factors accounted for a great part of the DEGs, while immune system signaling and cytokine signaling displayed the most significant changes. Furthermore, seven hub genes and nine transcription factors were identified. Interestingly, the top five upregulated DEGs were located on chromosome Y, and four of the top five downregulated DEGs were involved in immune and inflammation signaling. Further, the top DEGs were validated by RT-PCR in human samples. Our study explored the possible molecular mechanisms of heart failure caused by ischemic heart disease.

## 1. Introduction

Heart failure is a growing epidemic with substantial morbidity and economic burden. In modern society, coronary artery disease is the most common cause of heart failure. Patients with coronary heart disease, combined with hypertension, account for three-quarters of all heart failure patients [1]. Acute coronary artery obstruction leads to sharply reduced perfusion in the dominated ventricle wall. Myocardial necrosis results from insufficient perfusion, and hibernating myocardium in the vicinity directly causes acute cardiac dysfunction. Both symptomatic and asymptomatic coronary artery disease result in chronic heart ischemia, which can result in localized or diffuse myocardial fibrosis, heart enlargement and stiffness, reduced systolic and diastolic function, and eventually heart failure. This situation of irreversible heart dysfunction is termed ischemic cardiomyopathy (ICM) [2].

Ventricular remodeling is the basic mechanism of ischemic heart disease [3]. It is a process in which ventricular size, shape, and function are regulated by multiple factors, including neurohormonal factors [4], genetic background [5], mechanical stress [6], oxidative stress [7], and so on. The acute loss of myocardium brings about dramatic changes in heart stress and systemic hemodynamics, which activate the renin–angiotensin–aldosterone system (RAAS) and the sympathetic nervous system. Subsequently, the activation of various growth factors, including endothelin-1, fibroblast growth factor [8], platelet-derived growth factor [9], insulin-like growth factor, and transforming growth factor-β1 [10], induces adaptive myocyte hypertrophy and cardiac fibrosis. This process is accompanied by an increased volume intake through the kidneys. This vicious circle leads the damaged heart to overwork on a permanent basis. Myocardial infarction (MI) also induces macrophages, monocytes, and neutrophils to migrate into the infarct zone and release cytokines, which stimulate fibroblast proliferation, transform the interstitial fibroblast into myofibroblast [11], and further promote scar formation [12]. Although great efforts have been devoted to exploring the mechanism of ICM, a highly effective therapy directed against ICM is still lacking.

Coupled with the progress in sequencing and genomics, transcriptional signature analysis has proved powerful to provide insights into this disease. Hundreds of differentially expressed genes (DEGs) of heart failure have been obtained through several microarray profiling studies performed recently [13,14,15,16]. However, the results are inconsistent because of the sample heterogeneity in different studies and microarray platforms. In addition, human genomic studies in heart failure are constrained by insufficient heart samples from a single cohort study.

In order to identify the key genes or pathways associated with ICM, we comprehensively reanalyzed four microarrays of ICM from the gene expression omnibus (GEO) database (https://www.ncbi.nlm.nih.gov/geo/) collected in previous studies. In total, 255 common DEGs were found and further analyzed by gene ontology (GO) annotation, pathway enrichment, and protein–protein interaction analysis. We found that the immune system may be extremely critical in the process of ICM. These genes may help us to screen and identify novel biomarkers and treatment targets of ICM in the future.

## 2. Materials and Methods 

### 2.1. Data Source

The gene expression profiles were downloaded from the National Center for Biotechnology Information (NCBI) GEO database. Four data profiles were included: GSE5406, GSE26887, GSE42955, and GSE57338. Baseline information of each dataset is listed in Supplementary Table S1. We excluded samples with obvious age differences (age <20 and age >80).

### 2.2. Differential Expression Analysis

Four GEO Series were analyzed separately using online GEO2R with default parameters (https://www.ncbi.nlm.nih.gov/geo/geo2r/) [17]. The Benjamini and Hochberg false discovery rate method was selected for the adjustment of the *p*-values. NCBI-generated annotation was used to display the DEG list. Only the genes with an adjusted *p*-value less than 0.05 were considered as DEGs. The common DEGs among the four GSE datasets were screened by R programming language. *Log*(*FC_A_*) = *Log* (*a* + *b* + *c* + *d*)/4, where FC_A_ stands for average-fold change; *a* is fold change calculated from GSE5406 by GEO2R; *b* is fold change calculated from GSE26887; *c* is fold change calculated from GSE42955; *d* is fold change calculated from GSE57338. Quality control (including case selection and sample processing) was performed on the control group and ICM group in one dataset by the researchers who ran the microarrays, enabling cross-comparability. Since four datasets were from different research centers, there was inevitable group variation. It is unsuitable to conduct the data analysis on inter-datasets. Considering these aspects, we considered only the average value of Log FC that we obtained from each dataset to represent the expression level.

### 2.3. Gene Ontology Analyses

The PANTHER classifications database [18] was employed to perform the GO analysis, and pathway enrichment was analyzed by Reactome [19].

### 2.4. Protein–Protein Interaction Network Construction Analysis

The STRING online tools [20] and Cytoscape [21] were used to establish a protein–protein interaction (PPI) network, and the cutoff was that of the combined score >0.4. The network analyzer plug-in of Cytoscape software was used to analyze the topology property of the networks. Genes with edge degree >15 were defined as hub genes in this article.

### 2.5. Sample Collection

We included patients who were diagnosed with coronary heart disease by cardiac catheterization in our hospital and with left ventricle ejection fraction (LVEF) ≤40% [2]. The tissues for the validation of the DEGs were obtained in accordance with an ethics committee (Approval NO: B2014-084) at Zhongshan hospital, Fudan university, for research use. The samples were acquired from the left ventricle free wall of three unused donor hearts (control group) and three ICM patients with severe heart failure (New York heart association (NYHA) class IV). All samples were from men. The heart tissue was snap frozen in liquid nitrogen at the time of heart transplantation. The detailed documents of informed consent were reviewed by Fudan university, Zhongshan Hospital Review Board.

### 2.6. RNA Isolation and Real-Time Polymerase Chain Reaction 

The TRIzol (Invitrogen, Carlsbad, CA, USA) method was used to isolate total RNA. Then, RNA was reversely transcribed by using TransScript one-step genomic DNA (gDNA) removal (Thermo scientific, Vilnius, Lithuania) and a complementary DNA (cDNA) synthesis SuperMix kit (Thermo scientific), according to the manufacturer’s instructions. The expression of the top DEGs (Table 1) was analyzed on the Bio-Rad CFX connect real-time detection system (Bio-rad, Singapore, Singapore) with Power SYBR Green PCR Master Mix (TaKaRa, Shiga, Japan). The expression levels of 10 DEGs were normalized to glyceraldehyde-3-phosphate dehydrogenase (GAPDH). Relative gene expression levels were calculated using the 2-ΔΔCT (CT, cycle threshold) method. All primers are listed in Supplemental Table S4.

### 2.7. Statistical Analysis

The RT-PCR data was displayed as the mean ± SEM (standard error of mean) and analyzed by the two-tailed Student’s *t*-test. A value of *p* < 0.05 was considered statistically significant. All the experiments were repeated at least three times.

## 3. Results

### 3.1. Screening Common Differentially Expressed Genes from Four Gene Espression Omnibus Series

**Figure 1:**
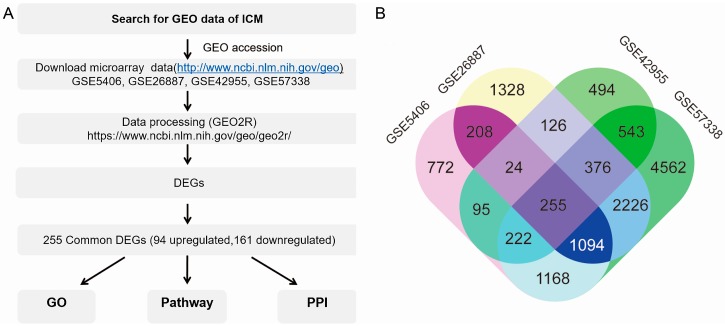
(**A**) Overall design of the study. (**B**) Identification of 255 commonly changed differentially expressed genes (DEGs) from four expression profile datasets (GSE5406, GSE26887, GSE42955, and GSE57338) using GEO2R. Different colored areas represent different datasets. The cross areas represent the commonly changed DEGs. Statistically significant DEGs were defined by *p* < 0.05. GEO: Gene expression omnibus; ICM: Ischemic cardiomyopathy; DEGs: Differentially expressed genes; GO: Gene ontology; PPI: Protein–protein interaction.

The overall design of this study is illustrated in Figure 1A. By searching for ICM of *Homo sapiens* in the GEO database, four series were finally included. Altogether, microarrays of the left ventricular myocardium from 159 control samples and 227 ICM samples were collected. Each GSE dataset was analyzed by GEO2R with default parameters. Four boxplots showed that the value data were normalized and median-centered across samples, which indicated that these microarray data were of high quality and cross-comparable (Supplemental Figure S1). There were 3840, 5639, 2137, and 10,488 DEGs with *p* < 0.05 in GSE5406, GSE26887, GSE42955, and GSE57338, respectively (Supplemental Table S1). Through screening by R programming language, 255 common DEGs were found (Figure 1B), including 93 (36.47%) upregulated DEGs and 162 (63.53%) downregulated DEGs (Supplemental Table S2). Among the 255 DEGs, the top five upregulated genes were *EIF1AY*, *UTY*, *USP9Y*, *RPS4Y1*, and *DDX3Y*. In addition, the top five downregulated ones were *SERPINA3*, *MYOT*, *S100A8*, *FCN3*, and *CD163*. These genes were listed in descending order according to fold change with their confirmed biological function (Table 1). Of note, the top five upregulated DEGs were located on chromosome Y. Except for *MYOT*, the other four of the top five downregulated DEGs were involved in immune and inflammation signaling. These results suggest that genes on chromosome Y and related to the immune system have a huge impact and could play vital roles in ischemic cardiomyopathy.

### 3.2. Functional Enrichment and Integrated Analysis of the Differentially Expressed Genes

**Figure 2:**
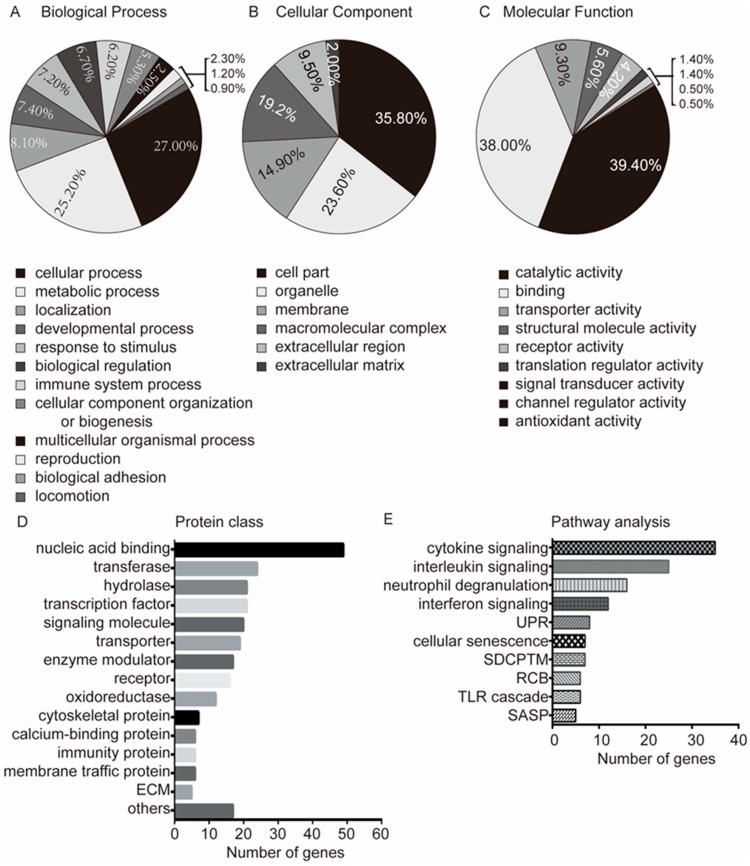
Gene ontology (GO) and pathway analysis of DEGs in ischemic cardiomyopathy. (**A**) Enriched GO terms in the biological process class; (**B**) Enriched GO terms in the molecular function class; (**C**) Enriched GO terms in the cellular component class; (**D**) The proteins of common DEGs were classified according to function; (**E**) Significantly enriched pathways of common DEGs. ECM: Extracellular matrix protein; UPR: Unfolded protein response; SDCPTM: SRP-dependent co-translational protein targeting to membrane; RCB: Regulation of cholesterol biosynthesis; TLR: Toll-like receptor; SASP: Senescence-associated secretory phenotype.

In order to precisely understand the changes in ICM genes, the GO of DEGs was analyzed with PANTHER database. The two most obvious changes in molecular function were catalytic activity (39.4%) and protein binding (38.0%), which were followed by transporter activity, structural molecular activity, receptor activity, translation regulator activity, and so on (Figure 2A). In terms of cellular components, the three most significant changes in ICM were found in the following classes: cell part (35.8%), organelle (23.6%), and membrane (14.9%). In addition, macromolecular complexes, extracellular region, and extracellular matrix displayed also significantly changes in ICM (Figure 2B). With regards to the biological process class, the DEGs were mostly clustered into cellular processes (27.0%) and metabolic processes (25.2%). In addition, other changes in biological processes in ICM were identified in localization, developmental processes, responses to stimulus, biological regulation, immune system processes, cellular component organization or biogenesis, multicellular organismal processes, reproduction, biological adhesion, and locomotion (Figure 2C).

To further comprehend the potential roles of DEGs, we also analyzed the protein classes of DEGs and found that the DEGs were mainly distributed among nucleic acid-binding proteins (19.7%), transferases (9.6%), hydrolases (8.4%), transcription factors (8.4%), signaling molecules (8.0%), transporters, enzyme modulators, receptors and oxidoreductases (Figure 2D). From the protein class results, the nuclear acid-binding proteins, enzymes, and transcription factors accounted for a great part of the DEGs. This result was consistent with the GO analysis (catalytic activity and protein binding) and suggested that these classes of proteins play important roles in ischemic cardiomyopathy.

### 3.3. Pathway Analysis

To gain a comprehensive insight into the pathways that are potentially important in ICM, we analyzed the pathways related to the 255 DEGs. Altogether, 62 pathways were obtained from Reactome. The immune system (61 DEGs involved), signal transduction, and metabolism were the three most obvious centers in the pathway network (Supplemental Figure S2). Among the top ten pathways (Figure 2E), five were involved in the immune system, including cytokine signaling, interleukins signaling, neutrophil degranulation, interferon signaling, and toll-like receptor cascade. Importantly, according to the number of DEGs involved, immune system pathways corresponded to the top four pathways, except for the toll-like receptor cascade. Meanwhile, two of the top ten pathways were involved in aging, i.e., cellular senescence and senescence-associated secretory phenotype (SASP). Moreover, the unfolded protein response (UPR) and the SRP-dependent cotranslational protein targeting to membrane (SDCPTM) were two of the top ten pathways related to changes in endoplasmic reticulum homeostasis. Also, the regulation of cholesterol biosynthesis (RCB) was present in the top ten pathways. This analysis suggested that immune system, lipid metabolism, and incorrect protein synthesis may be critical in the development of ICM. Genes involved in the top ten pathways are listed in Supplementary Table S3.

### 3.4. Protein–Protein Interaction Analysis

**Figure 3:**
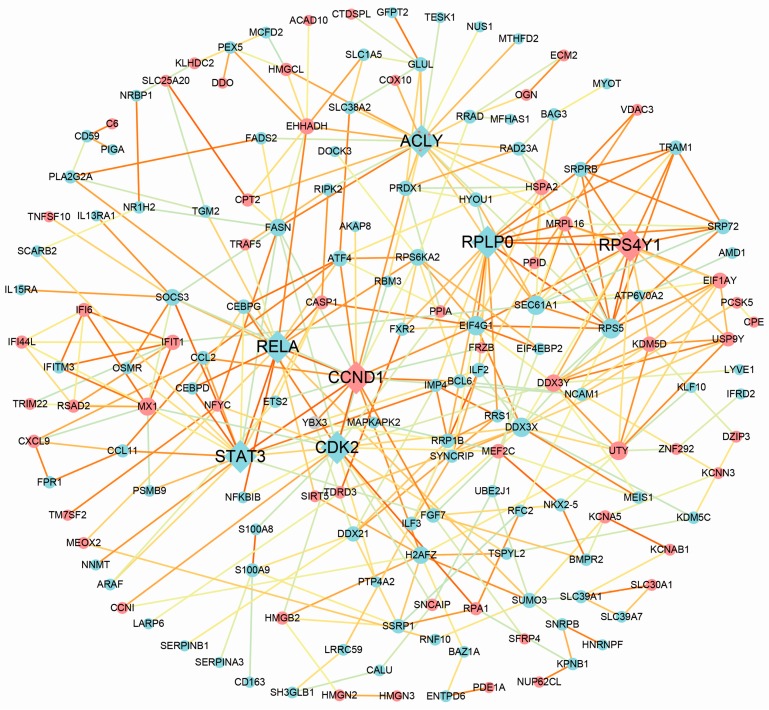
Protein–protein interaction (PPI) network complex. A total of 167 DEGs (113 upregulated genes and 67 downregulated genes) were filtered into the PPI network. Red nodes suggest upregulated genes and blue node suggest downregulated genes. The node size is proportional to the edge degree. The edge color suggests the significance according to the *p* value (the brighter the color, the smaller the *p* value).

Using the STRING online tool, we found 163 nodes with 347 protein–protein interaction (PPI) relationships, accounting for 63.2% of all the common DEGs (Figure 3). The properties of the network were analyzed, indicating that the network of PPIs was relatively aggregated (Supplemental Figure S3). Among the 163 nodes, 7 genes were identified as hub genes with the edge degree >15. According to the edge degree rank, the seven hub genes were *ACLY*, *CDK2*, *STAT3*, *CCND1*, *RPLP0*, *RPS4Y1*, and *RELA*. Except for *CCND1* and *RPS4Y1*, the rest of the five genes were all downregulated. The 7 hub genes could interact directly with 76 genes, and *ACLY* was the most intensive hub gene interacting with 6 upregulated genes and 15 downregulated ones in the network (Supplemental Figure S4). Interestingly, some hub genes could interact with multiple other hub genes. For example, *STAT3* could interact directly with three hub genes (*CDK2*, *CCND1*, and *RELA*), and *RPLP0* interacted with *ACLY* and *RPS4Y1* (Supplemental Figure S4). All the results suggested that the seven hub genes, especially *STAT3* and *RPLP0*, could play important roles in the development of ICM.

### 3.5. Real-Time Polymerase Chain Reaction Validation

**Figure 4:**
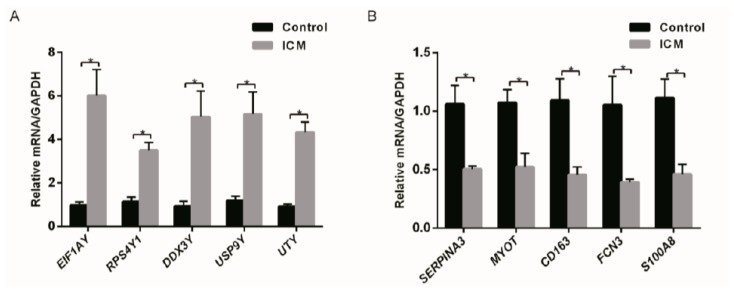
Relative expression level of DEGs. (**A**) *EIF1AY*, *RPS4Y1*, *DDX3Y*, *USP9Y*, and *UTY* were significantly upregulated in ICM patients. (**B**) *SERPINA3*, *MYOT*, *CD163*, *FCN3*, and *S100A8* were significantly downregulated in ICM patients (*n* = 3) compared to healthy controls (*n* = 3). * *p* < 0.05.

To ensure the reliability of the microarray results analysis, DEGs in Table 1 were selected randomly for validation. Real-Time PCR showed that *EIF1AY*, *UTY*, *USP9Y*, *RPS4Y1*, and *DDX3Y* were found to have a significantly higher expression (*p* < 0.05) in ICM heart samples than in normal ones. Meanwhile, *MYOT*, *S100A8*, *FCN3*, and *CD163* were significantly downregulated in ICM heart samples (Figure 4).

## 4. Discussion

ICM gradually progresses from coronary artery disease. High throughput research may facilitate the exploration of the critical mechanisms of ICM. Our study is the first comprehensive investigation focusing on expression profiling collected from microarray studies of ICM. It analyzed four gene expression profile datasets with 162 control samples and 227 ICM samples. Considering the different microarray platforms and analytical methods used in the four datasets, we reanalyzed the four datasets through GEO2R with a uniform standard for DEGs. In total, 255 common DEGs were obtained, which accounts for 0.88–0.15% of all genes in the different microarrays. With this large sample size, we have a good reason to believe that the 255 DEGs may play important roles in the occurrence and development of ICM. Through the basis of gene annotation databases, we further explored the possible mechanisms involved.

### 4.1. Inflammation and the Immune System Are Critical in the Development of Ischemic Cardiomyopathy

The principal finding of this study is that the immune system and inflammation signaling mediated by cytokines may play a vital role in the development of ICM. The impact of the immune system on the development of heart failure has been studied for years. Clearly, the involvement of the immune system is independent on the initial form of cardiac injury. Before the diffusion of transcriptomics studies, scientists noticed that a sustained activation of the adaptive immune system is a potential contributor to the progression of heart failure [22]. The innate inflammatory cascade, mediated largely by neutrophils and monocytes and macrophages [23], contributes to the chronic inflammation process. Recently, scientists have explained that the exosomes secreted by dendritic cells (DC) [24] and the DCs themselves were immunoprotective in the healing process of post myocardial infarction (MI) via the control of immune homeostasis [25]. The triggering of Toll-like receptors (TLRs) on DCs was critical for the functional maturation of DCs [26]. In our study, the Toll-like receptor cascade was significantly enriched in the pathway analysis (Figure 2E). In addition, *HLA-DPA1* and *HLA-DPB1*, which belong to the human leukocyte antigen (HLA) class II alpha chain and play vital roles in antigen presentation, were significantly upregulated, indicating the activation of dendritic cells in the heart. The imbalance between inflammatory and anti-inflammatory cytokines favors the onset of accelerated and extensive fibrosis [27]. Strategies to block the immune mediators, such as the cytokines and immunoglobulins, were developed to treat heart failure. None of them showed significant clinical improvements [28,29,30]. Targeting the dendritic cells may be a new approach to treat ICM.

Many aspects of the immune response pathway were also significantly changed in our study. First, four of the top five downregulated genes were involved in immune response signaling (Table 1) corresponding to several aspects of the immune response: immune response to elevated platelet cytosolic Ca^2+^ (*SERPINA3*); activated TLR4 signaling (*S100A8*); lectin pathway of complement activation (*FCN3*); scavenger receptors signaling (*CD163*). Secondly, the analysis of the pathways showed that half of the top ten significantly changed pathways of ICM were related to immune and inflammation signaling (Figure 2E) and contained 23.92% (61/255) of the DEGs. The effects of the mentioned DEGs and pathways with regards to the immune response in ICM may be of great interest for further study.

Our study provides supportive evidence for the fact that immune and inflammation factors are important in the later stage of ICM. In addition, it may provide new clues to identify the key targets and pathways in future research on the immunologic mechanisms of ICM.

### 4.2. The Improvement of Lipid Metabolism Could Be a Potential Target for Therapy of Ischemic Cardiomyopathy

A characteristic of late-stage heart failure is the impairment of myocardial fatty acid substrate metabolism. Under physiological conditions, free fatty acids undergoing β-oxidation are the main source of energy supply for the heart, comprising 40–60% of the energy supply. However, for ischemic heart disease, the energy preference switches to glucose. The decreased fatty acid oxidation and increased glucose metabolism result in heart damage due to lipotoxicity and acidosis. In the condition of severe ischemia, both sources of energy production are significantly impaired [31]. In our study, the expression of genes involved in cholesterol biosynthesis (Figure 2E), which include *NFYC*, *FASN*, *SEC24D*, *KPNB1*, *TM7SF2*, and *ACLY*, was significantly changed in ICM patients. *ACLY* (adenosine triphosphate (ATP) citrate lyase) and *FASN* (fatty acid synthase), two enzymes involved in fatty acid synthesis, had significantly low expression in ICM. *SEC24D* and *KPNB1* were also downregulated. Only *NFYC* and *TM7SF2* were upregulated. More importantly, *ACLY* was the most significant hub gene in the PPI analysis.

Mounting evidence suggests that this cluster of genes plays more extensive roles than energy metabolism. In cancer cells, inhibition of *ACLY* might affect both fatty acid elongation in the endoplasmic reticulum and fatty acid oxidation in the mitochondria [32]. Also, in skeletal muscle, the knockdown of *ACLY* in myotubes reduces the activity of mitochondrial complex I, IV, V, resulting in decreased ATP [33]. Moreover, it has been corroborated that the silencing of *ACLY* reduces histone acetylation in adipocytes, and *ACLY* activity links growth factor–induced energy metabolism to the regulation of histone acetylation [34]. *FASN* is the only lipogenic enzyme-coding gene in humans capable of synthesizing all important fatty acids de novo. Convincing evidence has accumulated documenting its multiple functions. *FASN* downregulation results in malonyl-coenzymeA(CoA) accumulation, which is responsible for hypoxic cell death in HepG2 cells [35]. A study in glioma showed that the inhibition of fatty acid synthase suppresses neovascularization by regulating the expression of vascular endothelial growth factor A (VEGF-A) [36]. Increased messenger RNA (mRNA) and protein expression of *FASN* was detected in human heart failure due to ICM, which is inconsistent with our result. In that study, only two patients were involved, and the samples were taken at the time of left ventricular assist device implantation. Substrate metabolism is unaffected in the heart of myocardium-specific *FASN* knockout mice. However, these mice are susceptible to sudden death under transverse aortic constriction [37]. The roles of *ACLY* and *FASN* in the development of ICM needs further study. *SEC24D* encodes a major subunit of COPII, which is a coat protein complex transporting hepatic ApoB100 containing lipoprotein from the endoplasmic reticulum (ER) to very low-density lipoproteins (vLDL) and contributes to the formation of vLDL [38]. *KPNB1* is important for the nuclear transfer of sterol regulatory element-binding proteins (SREBPs), which are key transcription factors for lipid synthesis [39]. *TM7SF2* is responsible for cholesterol biosynthesis and is regulated by SREBP-2 [40]. SREBPs interact with *NF-Y*, thereby synergistically boosting the transcription of the genes related to cholesterol and fatty acid metabolism [41]. Our study may provide some clues for further research regarding genes involved in lipid metabolism in the development of ICM.

### 4.3. Particular Genes on Chromosome Y May Be Risk Factors for Coronary Heart Disease and Ischemic Cardiomyopathy

One intriguing result of our study was that the top five upregulated DEGs were located on chromosome Y, including *EIF1AY*, *RPS4Y1*, *DDX3Y*, *USP9Y*, and *UTY*. Despite the gender bias in two of four datasets (GSE26887, GSE57338) which have fewer heart samples from female patients, we can still infer that the different expression of the five genes on chromosome Y is significant, the reason being that the five genes are also significant in the other two datasets in which there is no difference in sex ratio.

Previous studies have shown that the human chromosome Y is associated with increased risk of coronary artery disease [42]. Our work may agree, to an extent, with the findings of Charchar et al. [42]. In their study, different gene expression was found in the macrophages of men with haplogroup I on chromosome Y. Further analysis revealed that 19 molecular pathways showing strong differential expression between men with haplogroup I and other lineages of chromosome Y were interconnected by common genes related to inflammation and immunity, and that some of them had a strong relevance to atherosclerosis. In addition, another study by Bloomer et al. [43] showed that coronary artery disease-predisposing haplogroup I of chromosome Y was associated with the downregulation of *UTY* and *PRKY* genes in macrophage [43]. An expression profiling study based on new-onset heart failures caused by idiopathic dilated cardiomyopathy demonstrated that *USP9Y*, *DDX3Y*, *RPS4Y1*, and *EIF1AY* were significantly upregulated in male patients with high-fold changes [44]. The enigma between gender and coronary heart disease and ICM still needs further in-depth and systemic research based on epidemiological and basic studies. In the future, we may provide a platform for the gender-specific treatment of heart failure.

### 4.4. Could Heart Failure Induce Myocardial Regeneration?

Coronary artery disease leads to chronic heart failure, largely because the human heart has a limited potential to regenerate, as normal human cardiomyocytes renew from 1% annual turnover at the age of 25, to 0.45% at the age of 75 [45]. It has also been demonstrated that hypoxia induces heart regeneration in adult mice [46]. In rodent experiments, overexpressing the genes involved in heart development and growth to reprogram the non-cardiomyocyte into cardiomyocyte has been demonstrated to ameliorate heart function [47]. These genes are critical transcription factors in heart development, including *MEF2C*, *NKX2.5*, *GATA4*, and *HAND2*, among others. Unlike them, *MEIS1* is a critical transcriptional regulator of cardiomyocyte proliferation as its deletion was shown to prolong the postnatal proliferative window of cardiomyocytes in mouse heart. *MEOX2*, encoding the homeodomain transcription factor, is known to regulate endothelial cell proliferation and muscle development [48]. Research using loss-of-function approaches has shown that Meox2/Tcf15 haplodeficiency impaired the uptake of fatty acids by the endothelial cells of the heart and reduced fatty acid transfer to cardiomyocytes [49]. Nevertheless, its roles in ischemic heart disease have not been fully elucidated. In our study, *MEIS1* was downregulated in ICM, while *MEF2C* and *MEOX2* were upregulated. These could be interesting molecules in the research on myocardial regeneration.

### 4.5. Limitations of the Study

Although our analysis is powerful, with a high throughput and a large sample size, there are some limitations. Most of all, the number of genes represented in the Affymetrix Human Gene 1.0 ST Array (28,869 transcripts) is higher than that in the Affymetrix U133A Array (22,283 transcripts), which means that DEGs only detected in the last two datasets cannot be included in our overall analysis. Besides, none of the dataset is capable to detect the expression levels of noncoding RNAs, which have been declared to play critical roles in the development of heart failure [50]. Another limitation of this study is that there are inevitable confounding factors. There are less female samples in the ICM group in some datasets. Also, we did not further stratify the analysis by age. A previous study has shown that the expression level of genes is different in different gender and age groups [51]. However, age- and sex-matched heart samples are nearly impossible to obtain, not to mention that the morbidity of coronary artery disease in males is higher than in females. Despite these limitations, the large sample size of this study makes the results compelling.

## 5. Conclusions

In conclusion, we offer a novel and comprehensive analysis of gene expression profiles in failing hearts with ICM. Genes involved in inflammation and immune pathways, and genes on the Y chromosome were significantly changed in ICM. This analysis will provide valuable information for future research on the molecular mechanisms of ICM and offer clues for the discovery of novel therapeutic strategies.

## Figures and Tables

**Table 1:** The top 5 up- and down- regulated genes with confirmed biological function.

Gene symbol	Log(FC_A_)	Biological Function
*EIF1AY*	3.44	Chromatin organization
*UTY*	2.28	Chromatin organization
*USP9Y*	2.12	Ubiquitin-Proteasome Dependent Proteolysis
*RPS4Y1*	1.95	Chromatin organization
*DDX3Y*	1.78	Translational initiation
*SERPINA3*	−3.04	Immune Response to elevated platelet cytosolic Ca^2+^
*MYOT*	−2.27	Structural constituent of muscle
*S100A8*	−2.03	Immune System (Activated TLR4 signaling)
*FCN3*	−1.96	Immune System (Lectin pathway of complement activation)
CD163	−1.86	Scavenger Receptors signaling

FC_A_: average fold change of gene expression values; TLR4:Toll-like receptor 4.

## Supplementary Materials

The following are available online at http://www.mdpi.com/2073-4425/9/1/56/s1. Figure S1: Microarray quality control chart, Figure S2: Display of the overview of the pathway network from Reactome online, Figure S3: The topology property of the Cytoscape network, Figure S4: Network of seven hub genes, Table S1: Baseline information of samples from the four datasets, Table S2: List of the common DEGs of the four datasets, Table S3: List of DEGs involved in the top ten pathways, Table S4: Primer list of DEGs selected for RT-PCR.
