# Supplementary material for: Identification of a Common Different Gene Expression Signature in Ischemic Cardiomyopathy
Source: Genes (Basel). 2018 Jan 22;9(1):56. doi: 10.3390/genes9010056 (PMC5793207; doi:10.3390/genes9010056)
Supplement: Supplementary file 1 [file genes-09-00056-s001.pdf]

Supplementary Fig.S1 Microarray quality control chart.

Data after normalization. X-axis: sample; Y-axis: log_2 expression value. Box plot showed the distribution of value data for the samples we selected was median-centered, indicating the data are normalized and cross-comparable. A for GSE5406, B for GSE26887, C for GSE42955 and D for GSE57338.

Supplementary Fig.S2 Display of the overview of pathway network from Reactome online.

Overview of the pathway network of 255 DEGs generated by online Reactome tool. The immune system, signal transduction and metabolism with red circle are the three most enriched categories. Immune system is connected with signal transduction by several pathways.

Supplementary Fig.S3 The topology property of the Cytoscape network.

(A) Degree of distribution; The number of nodes is plotted on y-axis as the node degree on x-axis. The distribution of clustering coefficien decreases as the node degree increases. The number of genes decreased as the node degree inceased, reflecting the tendency to contain hub nodes. (B) Average clustering coefficient distribution; The average clustering coefficient is plotted on y-axis as a function of number of neighbours on x-axis. The distribution of clustering coefficien decreases as the node degree increases. The graph exhibits a decreasing tendency of average clustering coefficient as number of neighbours increases. The property follows the power law distribution and suggests a hierarchical organization of the network. (C) neighborhood connectivity distribution; The average neighbourhood connectivity is plotted on y-axis as number of neighbours on x-axis. The chart shows edges between low connected and highly connected nodes prevail in the network. (D) Topological coefficients; The distribution of topological coefficient is plotted on y-axis and the number of neighbours is plotted on x-axis. The graph indicates the tendency of the nodes in the network to have shared neighbors.

Supplementary Fig.S4 Network of seven hub genes

Hub gene located in the center of the network interacted with all the surrounding genes showed. Node size is related to proportional to the edge degree. Edge color suggests the significance according to p value (The brighter the color, the lower the p value). Red node suggests up-regulated genes and blue node suggests down-regulated genes.

Supplementary Table S1. Basline information of samples from the four datasets.

The characteristic of the subjects enrolled in our study is displayed, including: age, sex, the stage of heart failure. Sampling spot and the way of sample preservation and RNA isolation are listed. Con: non-ischemic heart with normal heart function; ICM: ischemic cardiomyopathy; LV: left ventricle; LVEF: left ventricle ejection fraction; NYHA: New York Heart Association (heart function classification). None: no information was available. #p>0.05, there was no significant difference of age between the control and ICM group in GSE 5406. * p<0.05, the difference of age between the control and ICM group was statistically significant in GSE26887 and GSE57338.

Supplementary Table S2. List of the common DEGs of the four datasets.

255 differentially expressed genes (DEGs) were identified from four profile datasets, including 93 up-regulated genes and 162 down-regulated genes in ICM compared to normal human heart sample. Genes were arranged according to fold change from the largest to the smallest. Genes in bold represent the key DEGs appeared in PPI network.

Supplementary Table S3. List of DEGs involved in the top ten pathways.

A number of pathways were significantly enriched in the Reactome network analysis (p<0.05). Here we clearly present the 10 most significant pathways, the number of genes involved and the gene symbol.

Supplementary Table S4. Primer list of DEGs selected for RT-PCR.

Fig. S1

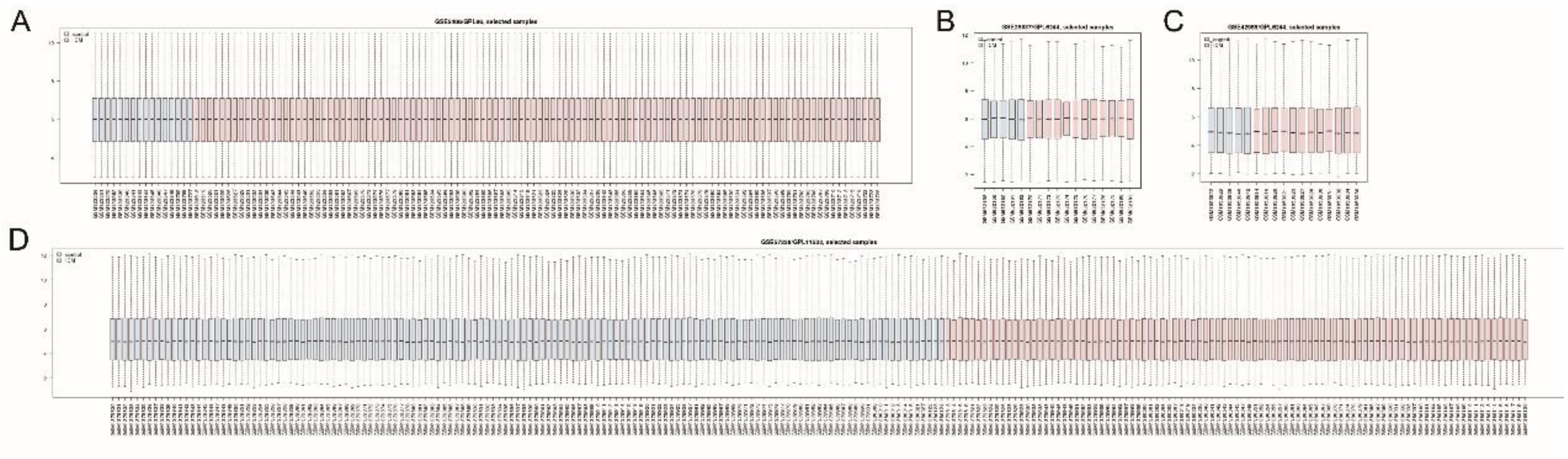

Fig. S2

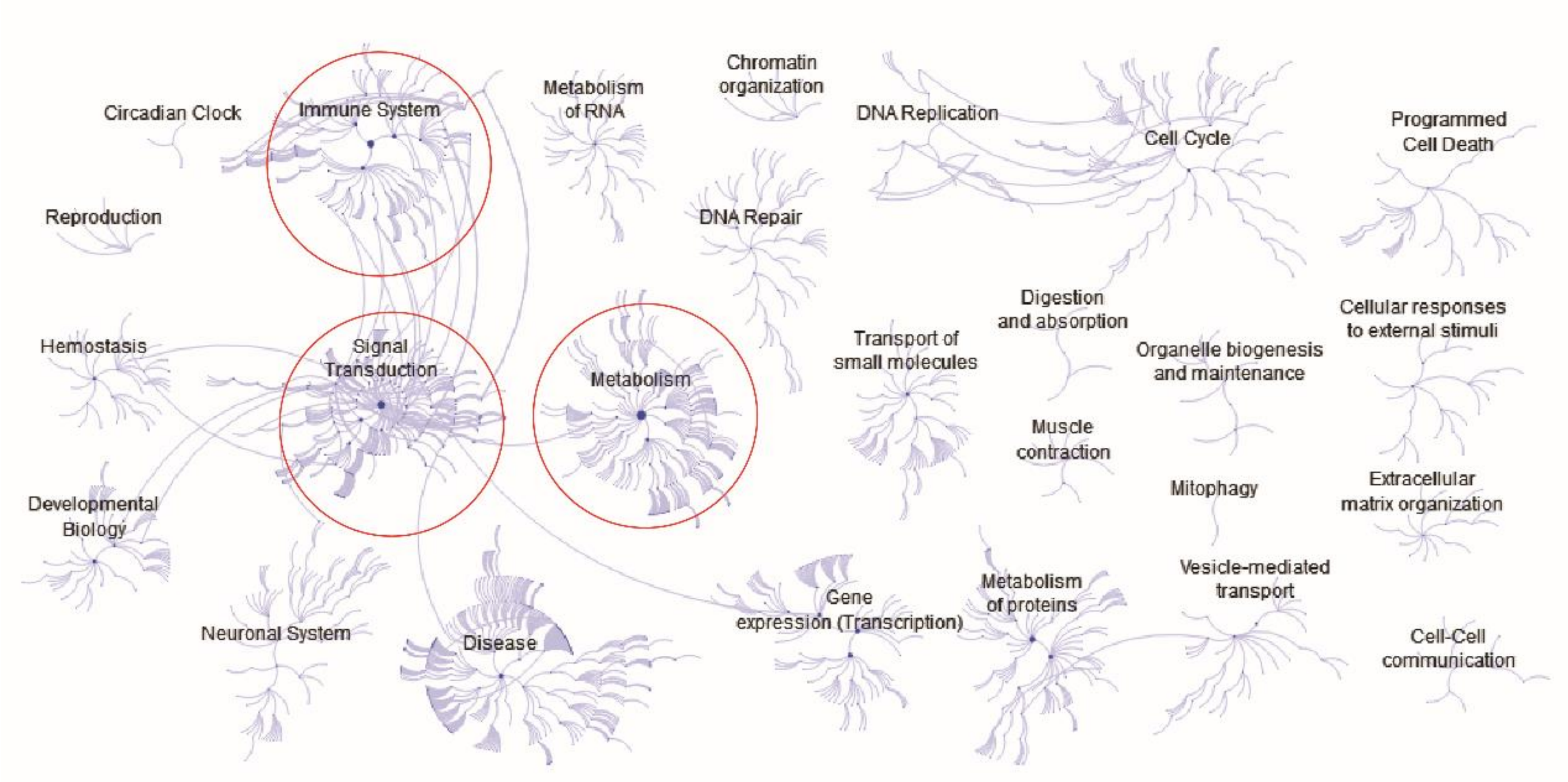
Circadian Clock
Immune System
Metabolism of RNA
Chromatin organization
DNA Replication
Cell Cycle
Programmed Cell Death
Reproduction
DNA Repair
Digestion and absorption
Cellular responses to external stimuli
Hemostasis
Signal Transduction
Metabolism
Transport of small molecules
Organelle biogenesis and maintenance
Muscle contraction
Mitophagy
Extracellular matrix organization
Developmental Biology
Neuronal System
Disease
Gene expression (Transcription)
Metabolism of proteins
Vesicle-mediated transport
Cell-Cell communication

Fig. S3

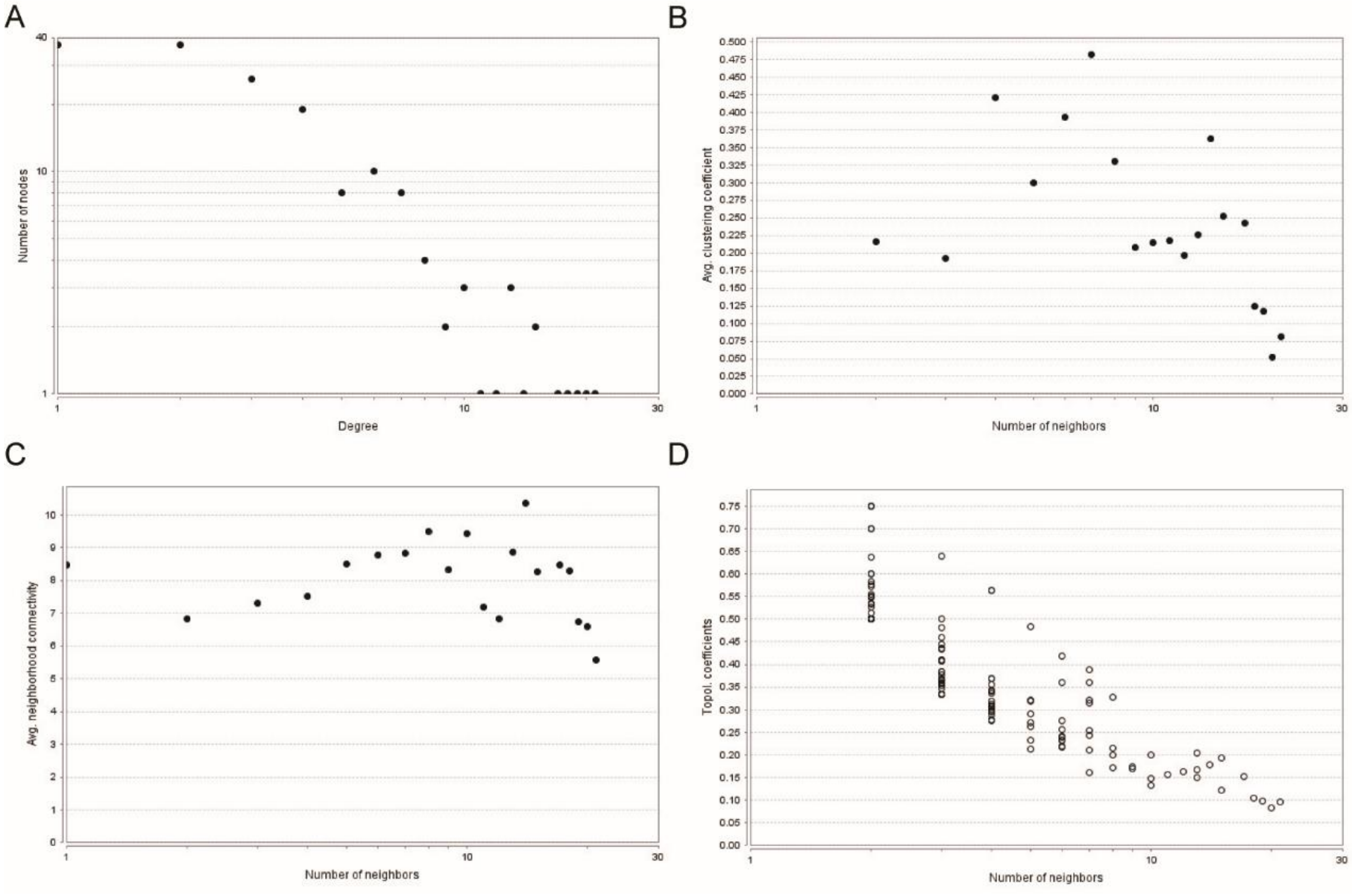
A
Number of nodes
Degree
B
Avg. clustering coefficient
Number of neighbors
C
Avg. neighborhood connectivity
Number of neighbors
D
Topol. coefficients
Number of neighbors

Fig. S4

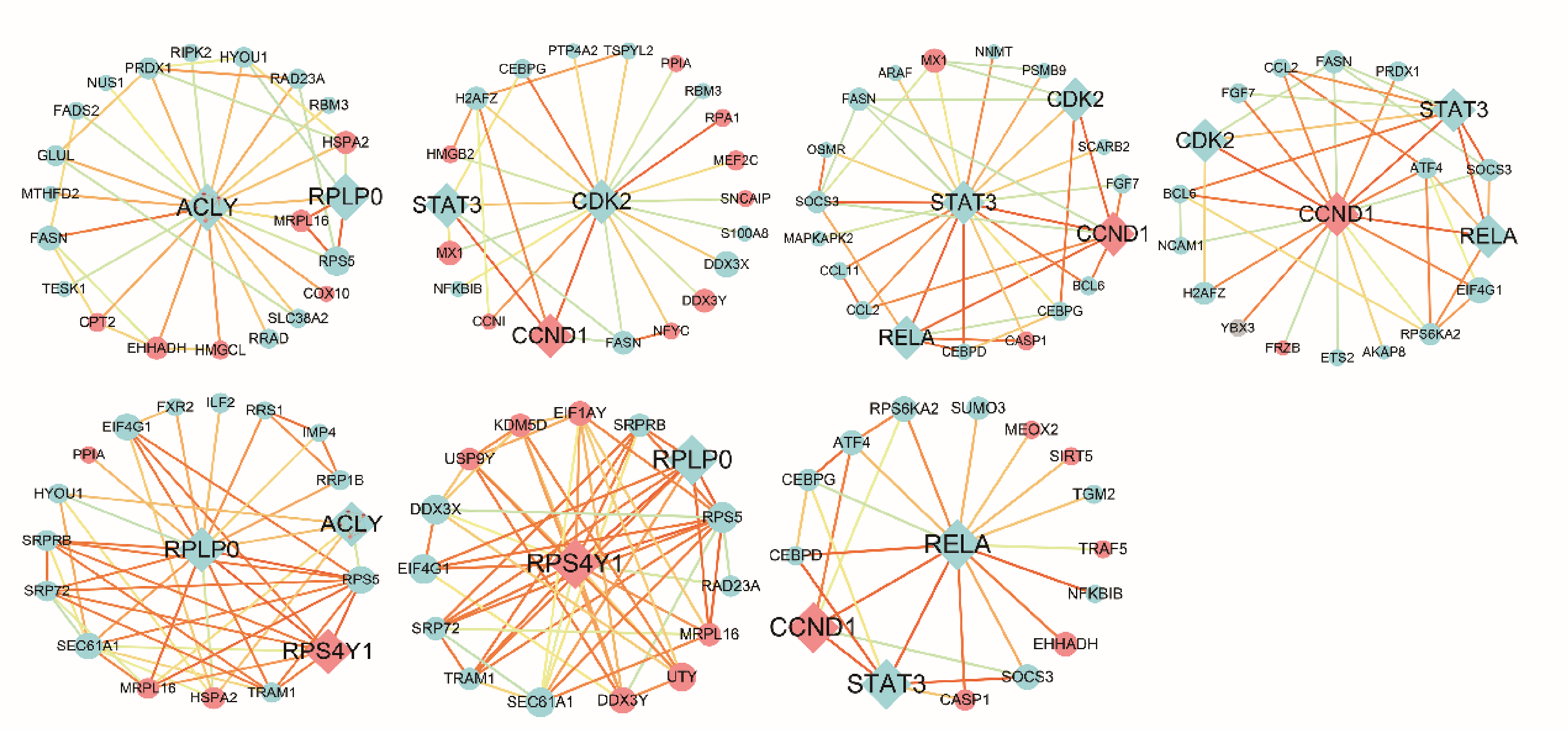

Table S1

	Number of Samples (n)	Cardiac area	Stage of heart failure	Age(year)	Male/female	Sample preservation	RNA isolation	Number of DEGs
GSE5406	Con (n=16)	Non-infarcted LV free wall	LVEF: 56±7%	(54±12)	None	snap-frozen in liquid nitrogen	Trizol (Invitrogen)	3840
	ICM (n=108)		NYHA classIII-IV LVEF: 14±8%	(57±12) #	None			
GSE26887	Con (n=5)	LV, vital and non-infarcted zone	normal LV function	48.4±1.2	2/3	snap frozen at -80°C	Trizol (Invitrogen)	5639
	ICM (n=12)		LVEF 27.6±1.9	60.4±4.3 *	12/0	Samples were immersed in RNAlater (QIAGEN GmbH) and stored at 4°C less than 24h before RNA extraction		
GSE42955	Con (n=5)	The apex of the LV	normal LV function	None	5/0	Stored at -80°C until the RNA extraction	Qiagen RNeasy Mini kit	2137
	ICM (n=12)		None		12/0			
GSE57338	Con (n=136)	LV free-wall tissue	normal LV function	49.4±1.3	74/62	Stored at -80°C until the RNA extraction	Qiagen RNeasy total RNA mini kit	10448
	ICM (n=95)		heart failure undergoing transplantation	59.1±0.7 *	81/14			

Table S2

93 Up- regulated Genes														
EIF1AY	*RPS4Y1*	*FRZB*	*DDX3Y*	*SFRP4*	*USP9Y*	*FAM162A*	*KDM5D*	*UTY*	*C6*	*PTN*	*HSPA2*	*ECM2*	*HMGN2*	*CPE*
JAM2	*ITIH5*	*HMGB2*	*LMO3*	*MEOX2*	*DZIP3*	*HLA-DPA1*	*AP3M2*	*HMGN3*	*TM7SF2*	*ARHGAP1*	*SLC25A20*	*CCND1*	*WFS1*	*KCNN3*
IFIT1	*IFI44L*	*CPT2*	*KCNA5*	*SLC30A1*	*SLC6A1*	*EHHADH*	*VDAC3*	*RAB3A*	*RSRP1*	*PDE1A*	*N6AMT1*	*SIRT5*	*EFHC1*	*PCSK5*
OGN	*TRAK1*	*KCNAB1*	*HMGCL*	*ACAD10*	*DDO*	*ZSCAN31*	*SH3TC2*	*RGN*	*ABHD11*	*DHRS11*	*TNFSF10*	*TRIM22*	*COX10*	*NFYC*
MRPL16	*MEF2C*	*MX1*	*TRAF5*	*SNCAIP*	*SEMA5A*	*HLA-DPB1*	*PYGB*	*CCNI*	*KLHDC2*	*ZNF277*	*SLC29A1*	*CNTRL*	*RPA1*	*CTDSPL*
GIMAP4	*IFI6*	*ACP6*	*IDS*	*CASP1*	*BTN3A3*	*NUP62CL*	*ZNF292*	*MAT2B*	*PPIA*	*TDRD3*	*TMCC1*	*VWA5A*	*ADAM18*	*RSAD2*
POFUT2	*RFX7*	*PPID*												
162 Down-regulated Genes														
SERPINA3	*MYOT*	*CCL2*	*CD163*	*FCN3*	*S100A8*	*PLA2G2A*	*STAT3*	*GLUL*	*LYVE1*	*SLC38A2*	*CEBPD*	*OSMR*	*GFPT2*	*NNMT*
MTHFD2	*S100A8*	*FASN*	*ETS2*	*KLHL41*	*PLP2*	*RARRES1*	*CCL11*	*LRRC59*	*YBX3*	*DDX3X*	*AMD1*	*SNRPB*	*UGCG*	*EMP3*
PTP4A2	*FPR1*	*BCL6*	*SOCS3*	*CALU*	*AASS*	*SLCO2A1*	*DDX21*	*RRAD*	*ATF4*	*MID1IP1*	*TSPYL2*	*SRPX*	*H2AFZ*	*CD59*
NCAM1	*RBM3*	*TGM2*	*RAMP1*	*SEC61A1*	*TRAM1*	*SERPINB1*	*EIF4G1*	*MCFD2*	*KIAA0040*	*SYNCRIP*	*SERPINB8*	*RPLP0*	*IFITM3*	*RRS1*
LARP6	*WDR1*	*SZRD1*	*RPS5*	*RIPK2*	*CEBPG*	*C6orf106*	*FGF7*	*PHTF2*	*SH3GLB1*	*BAG3*	*COBL*	*ACLY*	*SCARB2*	*SLC7A2*
RPS6KA2	*SLC1A5*	*ILF3*	*NPTX2*	*RAB9A*	*MEIS1*	*RNF126*	*BAZ1A*	*RAD23A*	*POPDC3*	*JTB*	*C1QTNF1*	*MFHAS1*	*ENSA*	*KIF1B*
NANS	*IFRD2*	*ILF2*	*IL15RA*	*SSRP1*	*UBE2J1*	*PRDX1*	*IL13RA1*	*SRP72*	*SLC39A1*	*IMP4*	*B4GALT5*	*TFE3*	*SBNO2*	*SLC39A7*
ARAF	*AKAP8*	*TESK1*	*SUMO3*	*NFKBIB*	*KPNB1*	*HYOU1*	*TMEM5*	*SEC24D*	*EHBP1L1*	*CDK2*	*HNRNPF*	*NR1H2*	*STEAP3*	*RELA*
CCDC6	*KDM5C*	*NRBP1*	*PIGA*	*NKX2-5*	*YRDC*	*MAPKAPK2*	*AKR1C1*	*FAM89B*	*SNX7*	*PEX5*	*LSM12*	*LRP4*	*FAM32A*	*NUS1*
PLEKHM2	*UBAP1*	*RFC2*	*TIMP4*	*FAM50A*	*RNF10*	*ATP6V0A2*	*RRP1B*	*DOCK3*	*KLF10*	*GTPBP2*	*HN1L*	*CHST3*	*FADS2*	*SRPRB*
PSMB9	*CXCL9*													

Table S3

Pathway name	Gene count	*p* Value	Genes involved
Cytokine Signaling	35	0.00035	*IFITM3 CCL11 CEBPD RPLP0 ARAF IFI6 FPR1 IFIT1 RELA RPS6KA2 FGF7 CCND1 SOCS3 CASP1 CCL2 HLA-DPA1 NCAM1 TRIM22 MTA2 IL13RA1 IL15RA; MEF2C RSAD2 STAT3 MX1; OSMR PSMB9 BCL6 HLA-DPB1 HNRNPF MAPKAPK2 RIPK2 PPIA KPNB1 EIF4G1*
Interleukins Signaling	25	0.00132	*CCL11 CEBPD RPLP0 ARAF FPR1 RELA SOCS3 FGF7 CCND1 RPS6KA2 CASP1 CCL2 NCAM1 MTA2 IL13RA1 IL15RA MEF2C RIPK2 STAT3 OSMR PSMB9 BCL6 HNRNPF MAPKAPK2 PPIA*
Neutrophil degranulation	16	0.01252	*DDX3Y PYGB SERPINA3 DDX3X RAB3A AMD1 FPR1 ILF2 ACLY SERPINB1 CD59 S100A9 PPIA S100A8 KPNB1 SLC29A1*
Interferon Signaling	12	0.01542	*IFITM3 SOCS3 HLA-DPA1 MX1 IFI6 NCAM1 IFIT1 TRIM22 KPNB1 HLA-DPB1 RSAD2 EIF4G1*
Unfolded Protein Response (UPR)	8	0.00002	*WFS1 TSPYL2 SEC61A1 NFYC SRPRB CCL2 HYOU1 ATF4*
SRP-dependent cotranslational protein targeting to membrane	7	0.01941	*TRAM1 SEC61A1 SRP72 RPS5 RPLP0 SRPRB PS4Y1*
Cellular Senescence	7	0.03672	*H2AFZ RPS6KA2 MAPKAPK2 CDK2 STAT3 ETS2 RELA*
Regulation of cholesterol biosynthesis	6	0.00354	*NFYC FASN SEC24D KPNB1 TM7SF2 ACLY*
Toll Like Receptor Cascade	6	0.02007	*MEF2C RPS6KA2 MAPKAPK2 RIPK2 RELA NFKBIB*
Senescence-Associated Secretory Phenotype (SASP)	5	0.01586	*H2AFZ RPS6KA CDK2 STAT3 RELA*

Table S4.

Primer list of DEGs selected for RT-PCR.

EIF1AY-	CCCACCTGCTGCATCTTAGTTC	SERPINA3-	AAAGTCACCAATCCCAAGCAAG
EIF1AY-	GCTTCCAATCGTCCATTTCCC	SERPINA3-	GGATAAGCAGACAGGGCCACAC
RPS4Y1-	TCGACAGGTCCCCACAAGC	MYOT-F	TTTCCCTATTCACTGCCCTTCC
RPS4Y1-	TGCGGTGAACAGCAAAACG	MYOT-R	TGTGCTTGCCTGTCCATCTACC
DDX3Y-	CTCATAAAGCGCAGGCACAC	CD163-F	CCCCACAAAAAGCCACAACAGG
DDX3Y-	AAGAACAGGTCCGCAGAAGG	CD163R	GCCAACAGAACAACCCCAAGGA
USP9Y-	GAAGAGCCTGCATTTCCACATAC	FCN3-F	CTGGCTGGGAAATGAGAAT
USP9Y-	CCACTCACAGCCTCATCCATAA	FCN3-R	CGCATAGTGGGCGAAAGTA
UTY-F	AACTAAGTGGTTGGGAGCGGG	S100A8-F	GTTTTTCAGGTGGGGCAAGTC
UTY-R	CATCTGGGAAACTACAGGGAAGG	S100A8-R	ACTGAGGACACTCGGTCTCT

References:

1. Zenz, R.; Eferl, R.; Scheinecker, C.; Redlich, K.; Smolen, J.; Schonthaler, H.B.; Kenner, L.; Tschachler, E.; Wagner, E.F. Activator protein 1 (Fos/Jun) functions in inflammatory bone and skin disease. *ARTHRITIS RES THER* **2008**, 10, 201.
2. Roy, A.; Srivastava, M.; Saqib, U.; Liu, D.; Faisal, S.M.; Sugathan, S.; Bishnoi, S.; Baig, M.S. Potential therapeutic targets for inflammation in toll-like receptor 4 (TLR4)-mediated signaling pathways. *INT IMMUNOPHARMACOL* **2016**, 40, 79-89.
3. Ishihara, S.; Schwartz, R.H. Two-step binding of transcription factors cause sequential chromatin structural changes at the activated IL-2 promoter. *J IMMUNOL* **2011**, 187, 3292-3299.
4. Thurlings, I.; de Bruin, A. E2F Transcription factors control the roller coaster ride of cell cycle gene expression. *Methods Mol Biol* **2016**, 1342, 71-88.
5. DeGregori, J.; Johnson, D.G. Distinct and overlapping roles for E2F family members in transcription, proliferation and apoptosis. *CURR MOL MED* **2006**, 6, 739-748.
6. Carvajal, L.A.; Hamard, P.J.; Tonnessen, C.; Manfredi, J.J. E2F7, a novel target, is up-regulated by p53 and mediates DNA damage-dependent transcriptional repression. *Genes Dev* **2012**, 26, 1533-1545.
7. Schoneveld, O.J.; Gaemers, I.C.; Lamers, W.H. Mechanisms of glucocorticoid signalling. *Biochim Biophys Acta* **2004**, 1680, 114-128.
8. Wu, B.; Wang, Y.; Wang, C.; Wang, G.G.; Wu, J.; Wan, Y.Y. BPTF is essential for T cell homeostasis and function. *J IMMUNOL* **2016**, 197, 4325-4333.
9. Hetz, C. The unfolded protein response: controlling cell fate decisions under ER stress and beyond. *Nat Rev Mol Cell Biol* **2012**, 13, 89-102.
10. Usui, M.; Yamaguchi, S.; Tanji, Y.; Tominaga, R.; Ishigaki, Y.; Fukumoto, M.; Katagiri, H.; Mori, K.; Oka, Y.; Ishihara, H. Atf6α-null mice are glucose intolerant due to pancreatic β-cell failure on a high-fat diet but partially resistant to diet-induced insulin resistance. *METABOLISM* **2012**, 61, 1118-1128.
11. Roychoudhuri, R.; Hirahara, K.; Mousavi, K.; Clever, D.; Klebanoff, C.A.; Bonelli, M.; Sciume, G.; Zare, H.; Vahedi, G.; Dema, B.; et al. BACH2 represses effector programs to stabilize T_{reg}-mediated immune homeostasis. *NATURE* **2013**, 498, 506-510.
12. Bernard, P.; Sim, H.; Knower, K.; Vilain, E.; Harley, V. Human *SRY* inhibits β-catenin-mediated transcription. *Int J Biochem Cell Biol* **2008**, 40, 2889-2900.
13. Cowan, J.; Tariq, M.; Ware, S.M. Genetic and functional analyses of *ZIC3* variants in congenital heart disease. *HUM MUTAT* **2014**, 35, 66-75.
14. Qiao, Y.; Giannopoulou, E.G.; Chan, C.H.; Park, S.H.; Gong, S.; Chen, J.; Hu, X.; Elemento, O.; Ivashkiv, L.B. Synergistic activation of inflammatory cytokine genes by interferon-gamma-induced chromatin remodeling and toll-like receptor signaling. *IMMUNITY* **2013**, 39, 454-469.
15. Harikumar, K.B.; Yester, J.W.; Surace, M.J.; Oyeniran, C.; Price, M.M.; Huang, W.C.; Hait, N.C.; Allegood, J.C.; Yamada, A.; Kong, X.; et al. K63-linked polyubiquitination of transcription factor IRF1 is essential for IL-1-induced production of chemokines CXCL10 and CCL5. *NAT IMMUNOL* **2014**, 15, 231-238.
